# Impaired Resolution of Inflammation in the *Endoglin* Heterozygous Mouse Model of Chronic Colitis

**DOI:** 10.1155/2014/767185

**Published:** 2014-07-10

**Authors:** Madonna R. Peter, Mirjana Jerkic, Valentin Sotov, David N. Douda, Daniela S. Ardelean, Niousha Ghamami, Flavia Lakschevitz, Meraj A. Khan, Susan J. Robertson, Michael Glogauer, Dana J. Philpott, Nades Palaniyar, Michelle Letarte

**Affiliations:** ^1^Molecular Structure and Function Program, The Hospital for Sick Children, Toronto, ON, Canada M5G 0A4; ^2^Department of Immunology, University of Toronto, Toronto, ON, Canada M5S 1A8; ^3^Keenan Research Centre in Li Ka Shing Knowledge Institute, St. Michael's Hospital, 209 Victoria Street, Toronto, ON, Canada M5B 1T8; ^4^Program in Physiology and Experimental Medicine, The Hospital for Sick Children, Toronto, ON, Canada M5G 0A4; ^5^Department of Laboratory Medicine and Pathobiology, University of Toronto, Toronto, ON, Canada M5S 1A8; ^6^Division of Rheumatology, Department of Pediatrics, The Hospital for Sick Children, Toronto, ON, Canada M5G 1X8; ^7^Department of Periodontology, Faculty of Dentistry, University of Toronto, Toronto, ON, Canada M5G 1G6; ^8^Institute of Medical Science, University of Toronto, Toronto, ON, Canada M5S 1A8

## Abstract

Endoglin is a coreceptor of the TGF-*β* superfamily predominantly expressed on the vascular endothelium and selective subsets of immune cells. We previously demonstrated that *Endoglin* heterozygous (*Eng* ^+/−^) mice subjected to dextran sulfate sodium (DSS) developed persistent gut inflammation and pathological angiogenesis. We now report that colitic *Eng* ^+/−^ mice have low colonic levels of active TGF-*β*1, which was associated with reduced expression of thrombospondin-1, an angiostatic factor known to activate TGF-*β*1. We also demonstrate dysregulated expression of BMPER and follistatin, which are extracellular regulators of the TGF-*β* superfamily that modulate angiogenesis and inflammation. Heightened colonic levels of the neutrophil chemoattractant and proangiogenic factor, CXCL1, were also observed in DSS-treated *Eng* ^+/−^ mice. Interestingly, despite increased macrophage and neutrophil infiltration, a gut-specific reduction in expression of the key phagocytic respiratory burst enzymes, NADPH oxidase 2 (Nox-2) and myeloperoxidase, was seen in *Eng* ^+/−^ mice undergoing persistent inflammation. Taken together, these findings suggest that endoglin is required for TGF-*β* superfamily mediated resolution of inflammation and fully functional myeloid cells.

## 1. Introduction

Chronic inflammation is characterized by prolonged leukocyte infiltration, dysregulated angiogenesis, and extensive fibrosis that contribute to tissue destruction. Defective resolution of inflammation has been implicated in several chronic diseases, including inflammatory bowel disease (IBD) [1]. Resolution is an active and complex process, involving several cellular and molecular mediators, that leads to suppression of proinflammatory responses and restoration of tissue structure and function [2]. Furthermore, immune-driven angiogenesis, caused by increased expression of proangiogenic cytokines and chemokines, is emerging as a major contributor to the development of persistent inflammation [3, 4].

The transforming growth factor beta (TGF-*β*) superfamily, which includes TGF-*β* isoforms, activins, and bone morphogenetic proteins (BMPs), is known to regulate many processes, including wound healing, angiogenesis, and immune responses [5–8]. TGF-*β*1, in particular, is a key regulator of inflammation resolution through direct effects on immune cells and surrounding stroma [8]. Indeed, dysregulation of TGF-*β* signaling pathway has been implicated in IBD patients [1]. TGF-*β* superfamily members bind to cell surface complexes of types I and II serine/threonine kinase receptors, which in turn phosphorylate intracellular Smad proteins, to mediate transcriptional control of specific genes [7]. In the present study, we determined whether the coreceptor endoglin (CD105), which modulates TGF-*β* superfamily responses [9], is involved in the regulation of inflammation.

Endoglin is a type I transmembrane glycoprotein primarily expressed by vascular endothelial cells [10]. Functionally, endoglin modulates TGF-*β* superfamily signaling pathways through interaction with ligand-binding receptors or direct binding of ligands [9, 11, 12]. Most notably, mutations in the* endoglin (ENG)* gene lead to the autosomal dominant vascular disorder, hereditary hemorrhagic telangiectasia type 1 (HHT1) [13]. HHT patients develop abnormal vascular structures, known as telangiectasia and arteriovenous malformations, which are unstable and prone to hemorrhage. The underlying mechanism of disease is haploinsufficiency and implicates endoglin as an important regulator of endothelial quiescence and blood vessel formation (angiogenesis) [14].

Endoglin is also present in activated monocytes, early mesenchymal and hematopoietic precursors, and some stromal cells, suggesting roles in immune responses and tissue repair [15–17]. Interestingly, a few studies reported increased incidence of severe infection in HHT patients [18, 19]. This increased susceptibility to infection suggests potential alterations in immune function in HHT patients. Indeed, impaired myelopoiesis and erythropoiesis have been observed in murine embryonic stem cells lacking endoglin [20]. Furthermore, HHT1-derived mononuclear cells were less abundant at sites of ischemic injury [21]. Thus, the function of endoglin in regulating TGF-*β* superfamily signaling and its expression in myeloid and stromal cells suggest involvement in inflammatory responses.

The* Endoglin *heterozygous (*Eng* ^+/−^) mouse is an experimental model of HHT1, while* Eng* null embryos die at mid-gestation of cardiovascular defects [22]. However, C57BL/6 congenic* Eng* ^+/−^ mice maintained in specific pathogen-free experimental animal facilities rarely show signs of HHT despite an underlying endothelial dysfunction associated with impaired vasomotor function [23, 24]. It was postulated that a second hit, such as an angiogenic or inflammatory stimulus, is necessary to induce disease. In a previous study, we challenged mice using the dextran sulfate sodium (DSS) model of experimental colitis and demonstrated that colitic* Eng* ^+/−^ mice show persistent epithelial ulceration, pathological angiogenesis, and increased leukocyte infiltration relative to colitic wild-type mice [25]. However, the mechanisms that led to the development of persistent inflammation and pathological angiogenesis remained to be explored.

Thus, we assessed whether expression of several TGF-*β* superfamily-related and angiogenesis-related genes was dysregulated in the colon of DSS-treated* Eng* ^+/−^ mice. In order to identify alterations in the immune response in* Eng* ^+/−^ mice, leukocyte subset distribution and number in the colon and bone marrow were also characterized. Our study revealed reduced colonic levels of active TGF-*β*1 in colitic* Eng* ^+/−^ mice, associated with lower expression of thrombospondin-1 (TSP-1), an angiostatic factor capable of activating TGF-*β*1 [26]. We demonstrated downregulated expression of the BMP- and activin-modulating factors, BMP endothelial cell precursor-derived regulator (BMPER), and follistatin (FST), which are known regulators of angiogenesis and inflammation [6, 27, 28]. Furthermore, increased expression of the proinflammatory and proangiogenic chemokine, CXCL1, was observed in DSS-treated* Eng* ^+/−^ mice. Interestingly, despite increased macrophage and neutrophil infiltration, reduced expression of the key phagocytic respiratory burst enzymes, NADPH oxidase 2 (Nox-2) and myeloperoxidase (MPO), was observed in colonic tissue of colitic* Eng* ^+/−^ mice. Overall, these findings suggest that endoglin is implicated in the resolution of inflammation.

## 2. Materials and Methods

### 2.1. Mice

*Eng* ^**+/**−^ mice were generated as previously described [22]. Congenic 14-15-week-old* Eng* ^**+/**−^ mice on the C57BL/6 background and control* Eng* ^**+/+**^ littermates were used. Mice were housed in a specific pathogen-free facility. All protocols were approved by the Animal Care Committee at the Toronto Center for Phenogenomics and the Hospital for Sick Children, in accordance with the Canadian Council on Animal Care.

### 2.2. DSS-Induced Colitis

As previously established, mice drank water supplemented with 3% DSS (DSS, m.w. 36,000–50,000; MP Biomedicals, Solon, OH) for 5 days and were then returned to normal water [25]. Body weight, water intake, activity, and diarrhea scores were measured daily for up to 23 days. At time of sacrifice, mice were anesthetized with ketamine (100 mg/kg intraperitoneally, i.p.) and xylazine (10 mg/kg i.p.) and perfused with phosphate-buffered saline through the left ventricle prior to organ harvesting. For gross histology, the distal colon was fixed in 4% paraformaldehyde, embedded in paraffin, sectioned longitudinally, and stained with hematoxylin and eosin. Representative images were obtained using the Olympus BX60 microscope (Center Valley, PA) at 100x magnification.

### 2.3. Cell Isolation

For colonic lamina propria (LP) cell isolation, 4 colons/genotype were pooled from day 0 and 3 colons/genotype were pooled from colitic mice. Colonic tissue was enzymatically digested to obtain a single cell suspension according to a previous protocol [29]. For total bone marrow cells, the femurs and tibias of each mouse were flushed with media, filtered through a 70 *μ*m strainer and red blood cells lysed. Cells were counted using trypan blue (Life technologies, Carlsbad, CA).

### 2.4. Flow Cytometry Analysis

Isolated cells were stained using the LIVE/DEAD fixable violet dead cell stain kit (Life technologies) followed by incubation with anti-mouse CD16/CD32 (eBioscience, San Diego, CA) to block Fc receptors. There were 3 different staining panels, with PE-TR anti-CD45 (Life technologies) present in all: (1) B cells (AF700 anti-CD19, eBioscience), (2) T cells (APC anti-CD3, FITC anti-CD4, and PE anti-CD8, BD Biosciences, San Jose, CA), and (3) myeloid cells (PerCP Cy5.5 anti-CD11b, PE-Cy7 F4/80, eBioscience; AF700 anti-Ly6C and FITC anti-Ly6G, BD Biosciences). Fluorescence-minus-one controls were also utilized. Samples were analyzed using the LSRII flow cytometer (BD Biosciences) and FlowJo software (Tree Star Inc., Ashland, OR). The total cell number per mouse was determined for each subset by multiplying the percentage of CD45^+^ leukocytes by the number of live cells counted after isolation.

### 2.5. Bacterial Composition and PCR Array

Total RNA was extracted from frozen colonic tissue using TRIzol reagent (Life technologies) and the Qiagen RNeasy Mini Kit (Hilden, Germany) with DNase I treatment.

For bacterial composition analysis, RNA was first converted to cDNA using Superscript VILO followed by real-time PCR analysis using Power SYBR green master mix (both Life technologies) and 16S rRNA primer sequences for total eubacteria,* C. coccoides* (XIVa),* C. leptum* (IV), and* Bacteroides* [30–32]. The results are presented as a relative abundance of total eubacteria.

Two different RT^2^ Profiler PCR arrays (Qiagen), the mouse angiogenesis array (PAMM-024A) and the TGF-*β*/BMP signaling pathway array (PAMM-035A), were used to profile mRNA expression. This data was analyzed using an Excel-based template provided by Qiagen.

### 2.6. Protein Measurements

Protein from frozen colonic tissues was extracted as previously described [25]. Protein from isolated cells was obtained using the CelLytic lysis reagent (Sigma-Aldrich, Oakville, CA) supplemented with complete protease inhibitors (Thermo Scientific, Rockford, IL).

Colonic TGF-*β*1 levels were measured using an R&D systems ELISA kit (DY1679, Minneapolis, MN). Total TGF-*β*1 was obtained by treating samples with 1 N HCl for 10 minutes followed by neutralization with 1.2 N NaOH/0.5 M HEPES, whereas endogenously active TGF-*β*1 was measured without acid treatment. The results are presented as pg of TGF-*β*1 per mg of protein.

Cytokine/chemokine profile in colonic tissues was measured using a Milliplex magnetic bead based immunoassay kit (Mouse Cytokine/Chemokine Panel I, EMD Millipore, St. Charles, MO) and analyzed using the Luminex 200 instrument (Austin, TX).

Western blot analysis was performed according to an established protocol [25]. The following antibodies were utilized: MPO (R & D Systems), Nox2 (BD Biosciences), TSP-1 (Thermo Scientific), and *β*-actin (Sigma-Aldrich). The enhanced chemiluminescence reagent from Perkin Elmer (Waltham, MA) was used for detection.

### 2.7. MPO Activity

The activity of MPO in colonic homogenates was measured by an established protocol [33]. One unit of MPO activity is defined as that degrading one micromole of hydrogen peroxide per minute at 25°C.

### 2.8. H_2_O_2_ and Superoxide Assays

H_2_O_2_ levels were measured in colonic homogenates using an Amplex Red assay (Life technologies) as previously described [34]. The effect of 100 *μ*M Apocynin (Sigma-Aldrich) was also tested. Fluorescence was quantified using 544 nm excitation and 590 nm emission, and H_2_O_2_ levels were normalized for protein content.

Bone marrow neutrophils were isolated as previously described [35]. The neutrophils were labeled for 15 minutes with 5 *μ*M dihydroethidium (Life Technologies), which reacts with superoxide to form ethidium which intercalates with DNA. Cells were stimulated with PMA (Sigma-Aldrich) for 30 minutes. The number of fluorescent nuclei was quantified using the CY3 fluorescence channel of the NIKON Cellomics ArrayScan VTI HCS Reader (Thermo Scientific). The results are presented as fold change in the number of fluorescent cells with PMA stimulation.

### 2.9. Neutrophil Migration

Isolated neutrophils were placed on a coverslip coated with 1% bovine serum albumin and incubated at 37°C for 15 minutes. The coverslip was placed on a Zigmond Chamber and neutrophils stimulated with 1 *μ*M fMLP (Sigma-Aldrich). The slide was placed under a Nikon Eclipse E400 microscope (Melville, NY) coupled with a Hamamatsu camera (Hamamatsu, Japan) and 4 frames/minute were captured over 15 minutes. The displacement and velocity of 330–390 neutrophils/genotype (3 experiments) were quantified using the Perkin Elmer Volocity software.

### 2.10. Statistical Analysis

Parametric data was assessed using the Student's* t*-test (2 groups) or one-way ANOVA (3 or more groups) followed by multiple comparisons using Tukey's post hoc testing. The Mann-Whitney test (2 groups) or the Kruskal-Wallis test (3 or more groups) with pairwise comparisons was utilized for nonparametric data. All statistical analysis, with exception of the PCR array data, was performed using the IBM SPSS software (Armonk, NY), and *P* < 0.05 was considered statistically significant.

## 3. Results

### 3.1. Persistent Colonic Inflammation and Altered Gut Microbiota Composition in DSS-Treated* Eng* ^+/−^ Mice

**Figure 1:**
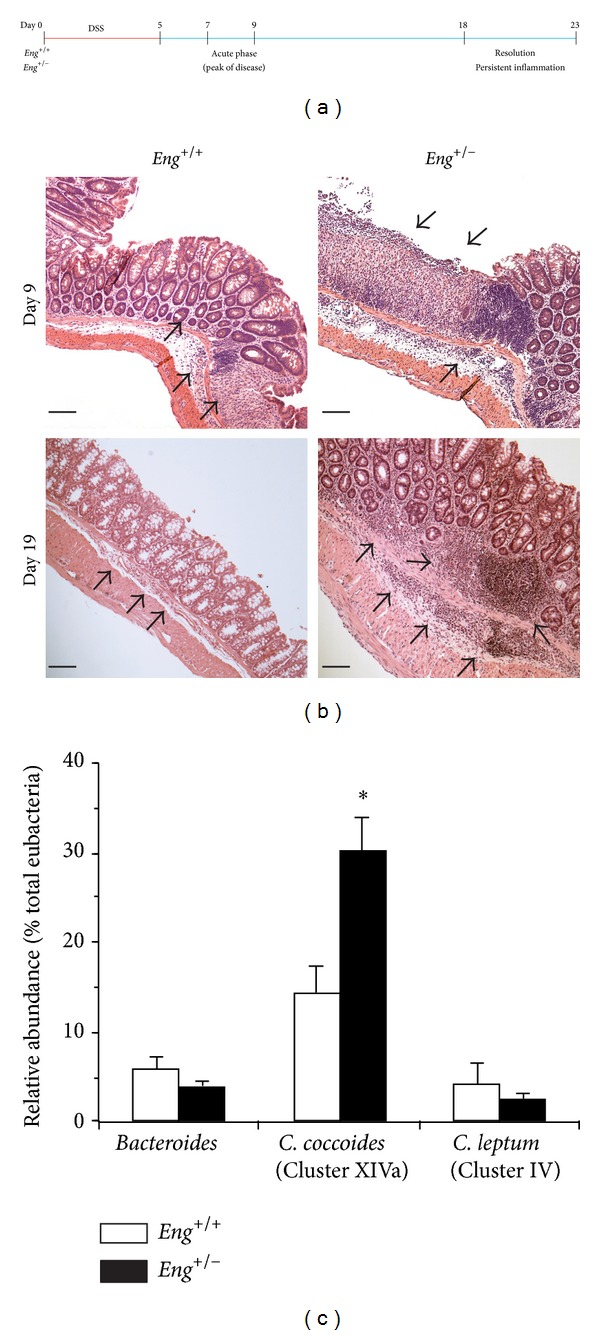
Persistent colonic inflammation in DSS-treated* Eng* ^+/−^ mice is associated with changes in a major gut bacterial group. (a)* Eng* ^+/+^ and* Eng* ^+/−^ mice were given a 3% DSS solution orally for 5 days (red), followed by return to normal drinking water (blue). The peak of inflammation occurred at days 7–9 for both genotypes; however, by days 18–23,* Eng* ^+/−^ mice showed signs of persistent inflammation, while* Eng* ^+/+^ mice were undergoing resolution of inflammation. (b) Representative hematoxylin and eosin stained distal colonic sections from colitic mice illustrate massive infiltration of leukocytes in the lamina propria (black arrows) in both* Eng* ^+/+^ and* Eng* ^+/−^ mice during the acute phase (day 9).* Eng* ^+/−^ mice show persistent leukocyte infiltration and incomplete crypt regeneration at day 19, whereas* Eng* ^+/+^ mice show minimal signs of inflammation. All images are at the same magnification (Bar = 100 *μ*m). (c) Relative abundance of various gut bacterial groups,* Bacteroides*,* Clostridium coccoides* (Cluster XIVa), and* Clostridium leptum* (Cluster IV), against total eubacteria in* Eng* ^+/+^ and* Eng* ^+/−^ mice at days 18–23 was determined by real-time polymerase chain reaction (PCR) using group-specific 16S rRNA primers. Results represent mean ± SEM (*N* = 4 mice for* Eng* ^+/+^group and 6 for* Eng* ^+/−^ group). *P < 0.05 versus corresponding* Eng* ^+/+^ group.

DSS acts as a chemical irritant when orally administered to mice and results in disruption of the gut epithelium leading to a robust inflammatory response accompanied by weight loss, diarrhea, bloody stools, and death in a percentage of animals [36]. We previously established [25] that providing 3% DSS in the drinking water for 5 days led to the development of severe colitis marked by extensive colonic epithelium damage that peaked in both* Eng* ^+/+^ and* Eng* ^+/−^ mice at days 7–9 and persisted in* Eng* ^+/−^ mice till days 18–23 while control mice recovered (Figure 1(a)). Days 7–9 represent the acute phase of colitis when both groups of mice demonstrate the highest weight loss, most severe diarrhea, and worse histological and inflammation scores.  Figure 1(b) (top panels) shows the extensive epithelial ulceration and immune cell infiltration observed in histological colonic sections from both groups of mice at day 9 of colitis. In* Eng* ^+/−^ mice, colonic inflammation and ulcerated epithelium persist while resolution of epithelial damage and inflammation is observed in* Eng* ^+/+^ mice, as shown by day 19 representative histological colonic sections (Figure 1(b), lower panels).

Endoglin haploinsufficiency may affect susceptibility to infection [19], and therefore, we assessed the relative abundance of various gut bacterial groups at days 18–23 of colitis. Group-specific primers were used to determine 16S rRNA levels of* Bacteroides*,* Clostridium coccoides* (Cluster XIVa), and* Clostridium leptum* (Cluster IV) [30–32]. No significant differences were observed between* Eng* ^+/+^ and* Eng* ^+/−^ mice in the* Bacteroides* and* C. leptum *groups; however, a higher relative abundance of* C. coccoides* was detected in colitic* Eng* ^+/−^ mice (Figure 1(c)). This increase in* C. coccoides* further illustrates the heightened disease severity in DSS-treated* Eng* ^+/−^ mice.

### 3.2. Altered Expression of Factors Regulating TGF-*β* Superfamily and Angiogenesis in Colitic* Eng* ^+/−^ Mice

**Figure 2:**
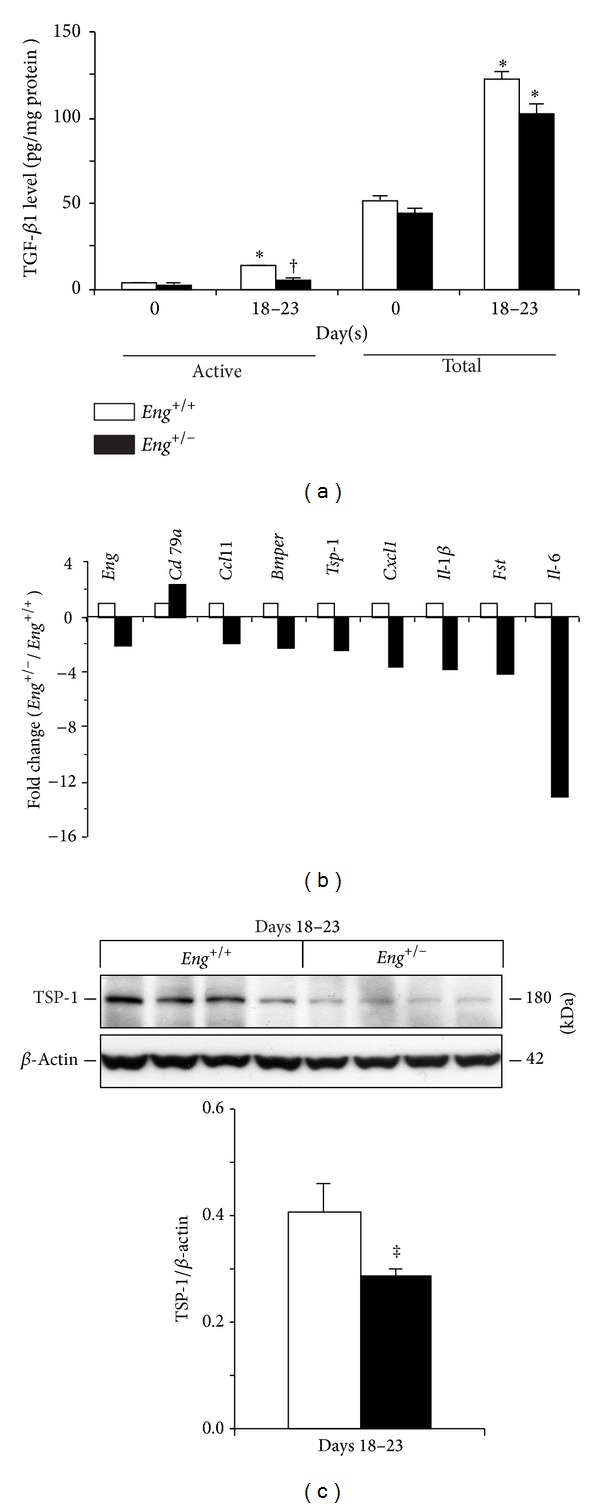
Reduced active TGF-*β*1 levels and altered expression of factors that regulate angiogenesis and resolution in* Eng* ^+/−^ mice undergoing persistent inflammation. (a) Endogenously active (without acid treatment) and total (with acid treatment) TGF-*β*1 levels were measured by ELISA in colonic tissue of* Eng* ^+/+^ and* Eng* ^+/−^ mice at days 0 and 18–23 (*N* = 6 mice for day 0 and 9-10 mice for days 18–23). ∗*P* < 0.05 versus corresponding day 0, ^†^ *P* < 0.05 versus corresponding* Eng* ^+/+^ group. (b) The mRNA expression profile of several angiogenic and TGF-*β* pathway-related genes was assessed in colons of* Eng* ^+/+^ and* Eng* ^+/−^ mice during the resolution phase. The results are expressed as a fold-change (*Eng* ^+/−^ over* Eng* ^+/+^ mice) and only significantly (*P* ≤ 0.05) altered genes with a 2-fold or greater change are shown (*N* = 4 mice for* Eng* ^+/+^ group and 5 for the* Eng* ^+/−^ group; BMP endothelial cell precursor-derived regulator (*Bmper*), follistatin (*Fst*), and thrombospondin-1 (*Tsp-1*)). (c) Representative immunoblot and densitometric analysis of TSP-1 expression, normalized to *β*-actin, in colons of* Eng* ^+/+^ and* Eng* ^+/−^ mice at days 18–23 of colitis. TSP-1 levels were nondetectable at days 0 and 7–9 (*N* = 6 mice for* Eng* ^+/+^ group and 5 for the* Eng* ^+/−^ group). ^‡^ *P* = 0.072 versus* Eng* ^+/+^ mice. Results represent mean ± SEM.

Given the importance of TGF-*β*1 in the resolution of inflammation [8], the levels of both endogenously active and total (active + latent) TGF-*β*1 were measured by ELISA in colonic tissue (Figure 2(a)). Under basal conditions (day 0, without DSS administration), active and total TGF-*β*1 levels were similar in mutant versus wild-typemice; however, active TGF-*β*1 levels were increased with colitis in wild-typebut not mutant mice. Colitis resulted in an increase in total TGF-*β*1 levels in both* Eng* ^+/+^ and* Eng* ^+/−^ mice, but with no significant difference between the genotypes.

In order to understand the mechanisms that lead to persistent inflammation in* Eng* ^+/−^ mice, the mRNA expression profile of several angiogenic and TGF-*β* superfamily pathway-related genes was assessed in the colon of DSS-treated mice during the resolution phase. With the exception of CD79A, a B cell receptor component, all other significantly altered factors were downregulated in colitic* Eng* ^+/−^ mice (Figure 2(b)). As expected,* Eng* ^+/−^ mice expressed half the endoglin mRNA levels compared to wild-type mice. Downregulated expression of BMPER, an extracellular regulator of various BMPs and modulator of angiogenesis and endothelial activation [27, 28], was also observed in colitic* Eng* ^+/−^ mice. In addition, persistent inflammation in* Eng* ^+/−^ mice was associated with reduced expression of FST, an antagonist of activin-mediated proinflammatory responses and a promoter of wound healing [6, 37]. Lower mRNA (Figure 2(b)) and protein (Figure 2(c)) expression of the angiostatic factor thrombospondin-1 (TSP-1), also known as an activator of latent TGF-*β*1 [26], were observed in colitic* Eng* ^+/−^ mice. TSP-1 expression was not detectable in control mice or during the acute phase. Thus, the decrease in TSP-1 may be responsible for the lower levels of active TGF-*β*1 and contribute to the increased angiogenesis in colitic* Eng* ^+/−^ mice.

In addition, the mRNA expression of several cytokine/chemokine (IL-6, CXCL1, CCL11, and IL-1*β*) genes was low in* Eng* ^+/−^ mice at days 18–23 of colitis (Figure 2(b)). Many of these factors regulate myeloid responses. Thus, persistent disease in* Eng* ^+/−^ mice is associated with reduced active TGF-*β*1 production and dysregulated expression of factors modulating immune responses, angiogenesis, and tissue repair.

### 3.3. Increased CXCL1 Levels in the Colon of DSS-Treated* Eng* ^+/−^ Mice in the Acute Phase of Disease

Many cytokines/chemokines have been implicated in the DSS model of experimental colitis [36]. Therefore, we measured the protein levels of these factors in the colon of control and colitic mice using Milliplex multianalyte profiling (Table 1). All factors were low or below detection at day 0 (baseline) in both* Eng* ^+/+^ and* Eng* ^+/−^ mice. At the peak of disease (days 7–9), while both groups demonstrated increased levels of myeloid-regulating factors, heightened expression of the neutrophil chemoattractant, CXCL1 (KC) [38], was found in* Eng* ^+/−^ mice. By the resolution phase (days 18–23), both genotypes exhibited a low level of expression of CXCL1, G-CSF, IL-6, and CCL11. The observed lower mRNA levels of these factors are likely the result of a negative feedback loop to suppress the enhanced inflammatory response in DSS-treated* Eng* ^+/−^ mice. We also show enhanced CXCL1 expression in the acute phase in* Eng* ^+/−^ mice, suggesting that heightened inflammation is already occurring in these mice.

### 3.4. Colitic Mice Show Increased Myeloid Cell Infiltration in Colonic Lamina Propria

To investigate potential alterations in the immune response of* Eng* ^+/−^ mice, the distribution (expressed as % CD45^+^ leukocytes) and number of total T, B, and myeloid cells isolated from colonic LP were analyzed by flow cytometry in control and DSS-treated mice.

The proportion of total B (CD19^+^) lymphocytes, estimated at 26–35% of leukocytes, was unchanged after colitis induction and between genotypes (Table 2). However, by the resolution phase, there was a similar increase in total B cell number in both groups. Therefore, the increased expression of CD79A in the colon of* Eng* ^+/−^ mice may represent heightened B cell activation.

The percentage of T lymphocytes (CD3^+^ cells) was maintained at ~13–16% before and during colitis in both genotypes, while their total number increased by days 18–23 of colitis in both groups of mice (Table 2). The majority of T cells, regardless of DSS treatment and genotype, were CD4^+^, while the remaining were mostly CD8^+^ cells. Furthermore, the lack of increase in Th1, Th2, and Th17 cytokines (Table 1) further emphasizes that T cells were not the major contributor to disease in DSS-treated* Eng* ^+/−^ mice.

Myeloid cells (CD11b^+^) were present in the colon under noninflammatory conditions and at the same frequency (~19–22%) in* Eng* ^+/+^ and* Eng* ^+/−^ mice (Table 2). During the acute phase, the overall myeloid distribution was significantly expanded in* Eng* ^+/+^ mice and showed a trend in* Eng* ^+/−^ mice. By the resolution phase, the proportion of myeloid cells declined in* Eng* ^+/+^ mice but remained somewhat higher in* Eng* ^+/−^ mice (*P* = 0.08 between days 0 and 18–23). Both groups of mice showed higher total numbers of myeloid cells at days 18–23 of colitis compared to day 0, with a trend for more CD11b^+^ cells in* Eng* ^+/−^ mice.

### 3.5. DSS-Treated* Eng* ^+/−^ Mice Show Higher Numbers of Colonic Macrophages

**Figure 3:**
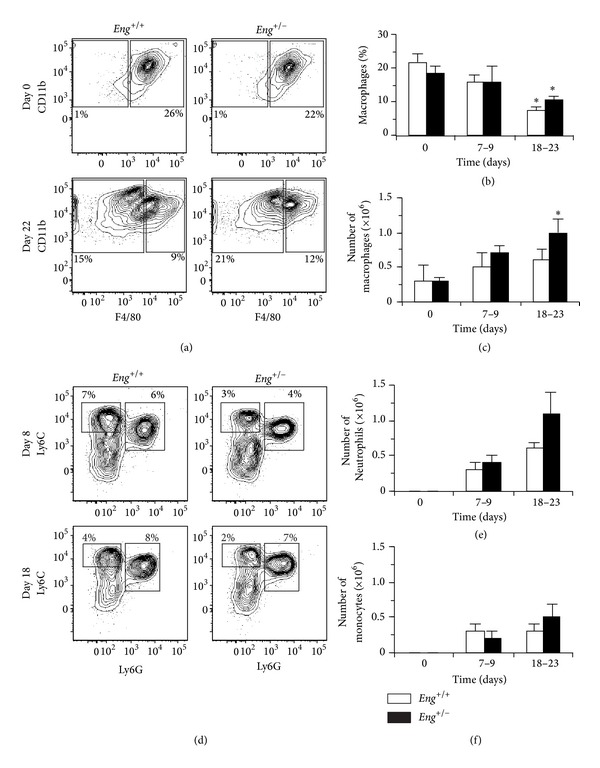
Higher number of infiltrating myeloid cells in colonic lamina propria of colitic* Eng* ^+/−^ mice. (a) Representative flow cytometry contour plots of colonic lamina propria cells, isolated at day 0 and day 22 of a DSS-induced colitis experiment, show the distribution of CD11b^+^F4/80^+^ and CD11b^+^F4/80^−^ cells. All plots were first gated for CD45^+^CD11b^+^ cells (not shown) and the percentage of CD45^+^ leukocytes is indicated. (b) The distribution and (c) total number of CD11b^+^F4/80^+^ cells in* Eng* ^+/+^ and* Eng* ^+/−^ mice at days 0, 7–9, and 18–23. (d) Representative flow cytometry contour plots of colonic lamina propria cells isolated from both groups of mice at days 8 and 18 of the colitis course. Cells were initially gated for CD11b^+^F4/80^−^ cells and analyzed for neutrophils (Ly6C^+^/^−^Ly6G^+^) and monocytes (Ly6C^+^Ly6G^−^). Histograms in (e) and (f) show the number of neutrophils and monocytes per mouse, respectively. Results represent mean ± SEM (*N* = 3 experiments for day 0, 5 for days 7–9, and 4 for days 18–23, with 3-4 mice/experiment). *P < 0.05 versus corresponding day 0.

Given the increased levels of myeloid recruiting/activating factors in colitic mice, we examined these subsets within the CD11b^+^ compartment. Prior to DSS induction, the LP myeloid cells of* Eng* ^+/+^ and* Eng* ^+/−^ mice were predominantly CD11b^+^F4/80^+^ macrophages (Figure 3(a)). The percentage of macrophages was unchanged at the peak of disease compared to day 0 but was significantly reduced by the resolution phase in both genotypes (Figure 3(b)). However, the total number of macrophages per mouse increased by days 18–23 in* Eng* ^+/−^ but not* Eng* ^+/+^ mice (Figure 3(c)).

Upon DSS induction, CD11b^+^F4/80^−^ cells became the predominant myeloid population in both genotypes (Figure 3(a)). The representative flow contour plots of CD11b^+^F4/80^−^ cells show neutrophils (Ly6C^+/−^Ly6G^+^) and monocytes (Ly6C^+^Ly6G^−^) as the major myeloid subsets infiltrating the colon in both groups of mice throughout colitis (Figure 3(d) **)**. Neutrophils were not detected prior to colitis but varied in distribution (from 4% to 15% of total leukocytes) after DSS induction in both genotypes. However, a trend for higher neutrophil numbers was observed in* Eng* ^+/−^ mice at days 18–23 (Figure 3(e)). The distribution and number (Figures 3(d) and 3(f)) of monocytes were similar between genotypes. Interestingly, there was a population of CD11b^+^F4/80^−^Ly6C^−^Ly6G^−^ cells (2–4% of leukocytes during colitis in both groups) that may represent an immature myeloid subset (Figure 3(d)). This subset increased to similar numbers in both genotypes during colitis (from 0.01 × 10^6^ cells/mouse at day 0 to 0.2-0.3 × 10^6^ cells/mouse during colitis). Thus, our findings suggest that enhanced infiltration of myeloid cells in colon of DSS-treated* Eng* ^+/−^ mice is associated with increased inflammation.

To determine whether myeloid cells are intrinsically defective in* Eng* ^+/−^ mice, we examined these subsets in their bone marrow using flow cytometry. At the peak of colitis, the total neutrophil number declined at a similar level in both genotypes (9.5–9.7 × 10^6^ cells/mouse at day 0 to 5.4 × 10^6^ cells/mouse at days 7–9), suggesting normal exit from the bone marrow of* Eng* ^+/−^ mice. By the resolution phase, a similar increase in the number of neutrophils (28–30 × 10^6^ cells/mouse) was detected in both groups. Macrophages initially increased in number (2-3 × 10^6^ cells/mouse at day 0 to 4-5 × 10^6^ cells/mouse at days 7–9); however, these numbers normalized to basal levels at days 18–23 in both genotypes. Thus, generation and exit of myeloid cells from the bone marrow during inflammation are normal in* Eng* ^+/−^ mice.

### 3.6. *Eng* ^+/−^ Mice Exhibit Lower Colonic MPO and Nox-2 Levels during DSS-Induced Colitis

**Figure 4:**
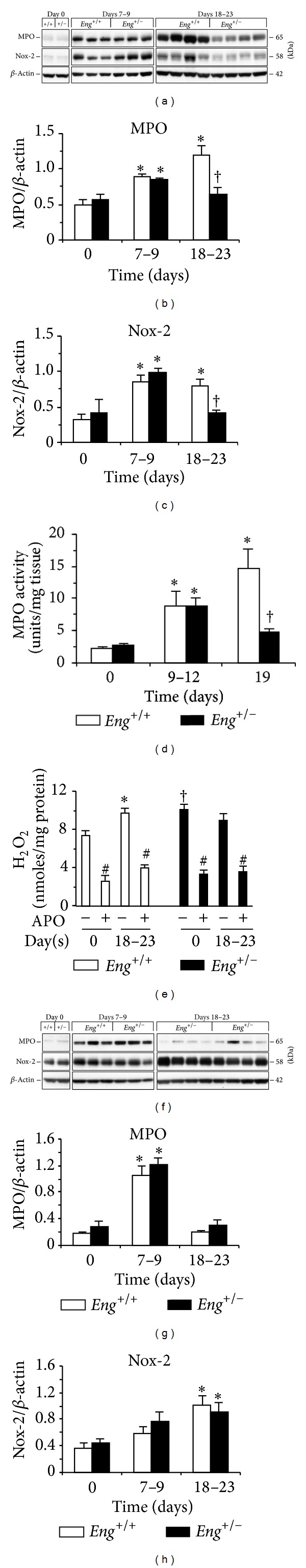
Reduced colonic MPO and Nox-2 expression in colitic* Eng* ^+/−^ mice are not associated with an intrinsic bone marrow defect. (a) Representative Western blots show colonic expression levels of MPO and Nox-2 in basal and colitic mice, normalized to *β*-actin (days 0 and 18–23 images are derived from a single immunoblot). Histograms illustrate densitometric analysis of (b) MPO and (c) Nox-2 expression (for MPO: *N* = 6–8 mice for day 0 and 4–9 mice for days 7–9 and 18–23; for Nox-2: *N* = 4-5 mice for day 0, 3–7 for days 7–9 and 18–23). (d) Colonic MPO enzymatic activity was assessed in* Eng* ^+/+^ and* Eng* ^+/−^ mice at days 0, 9–12, and 19 of colitis (*N* = 6–8 mice for days 0, 9–12, and 19). (e) The H_2_O_2_ levels in colons of mice at days 0 and 18–23 were measured using an Amplex Red assay. Apocynin (APO) was utilized as an NADPH oxidase inhibitor (*N* = 6–8 mice for day 0 and 4–7 for days 18–23). (f) Total bone marrow cells were isolated and MPO and Nox-2 expression was assessed using Western blot analysis. Representative images show bone marrow expression levels of MPO and Nox-2 in basal and colitic* Eng* ^+/+^ and* Eng* ^+/−^ mice, with *β*-actin as the normalizing factor (days 0 and 18–23 images are derived from a single immunoblot). Histograms illustrate densitometric analysis of (g) MPO and (h) Nox-2 expression at all-time points tested (for MPO and Nox-2: *N* = 3 mice for day 0 and 3–5 mice for days 7–9 and 18–23). *P < 0.05 versus corresponding day 0, ^†^ *P* < 0.05 versus corresponding* Eng* ^+/+^ group, and ^#^ *P* < 0.05 versus corresponding untreated group. Results represent mean ± SEM.

MPO and the phagocyte Nox-2 are two important enzymes that regulate reactive oxygen species- (ROS-) mediated bacterial clearance. Western blot analysis showed that MPO and Nox-2 levels were increased in both groups at days 7–9; however, by days 18–23, lower levels of these enzymes were found in* Eng* ^+/−^ compared to* Eng* ^+/+^ mice (Figures 4(a) and 4(c)). Colonic MPO enzymatic activity was also lower at days 18–23 in* Eng* ^+/−^ mice, likely representing reduced overall expression in the tissue (Figure 4(d)). ROS production was analyzed by measuring colonic hydrogen peroxide (H_2_O_2_) levels, a downstream by product of increased ROS (Figure 4(e)) [39]. Prior to DSS induction, H_2_O_2_ levels were significantly higher in the colons of* Eng* ^+/−^ mice, likely due to uncoupling of endothelial nitric oxide synthase (eNOS) and generation of eNOS-derived ROS as shown previously [34]. In the current study, we observed increased H_2_O_2_ levels with DSS colitis in* Eng* ^+/+^ mice but not in* Eng* ^+/−^ mice, when comparing days 0 to 18–23. Most of the H_2_O_2_ production was dependent on NADPH oxidase activity, as ROS production was decreased by 60–65% by the Nox inhibitor apocynin [40]. Thus, the decrease in MPO and Nox-2 expression associated with persistent colitis in* Eng* ^+/−^ mice may lead to impaired ROS mediated bacterial clearance.

To determine whether there was any intrinsic defect in MPO and Nox-2 expression, bone marrow cells were isolated from control and colitic mice and analyzed. An increase in MPO was observed at days 7–9 in both genotypes (Figures 4(f) and 4(g)). In contrast, Nox-2 expression was upregulated at days 18–23 but to the same level in both genotypes (Figures 4(f) and 4(h)). Therefore, the defects in MPO and Nox-2 in* Eng* ^+/−^ mice were gut specific.

**Figure 5:**
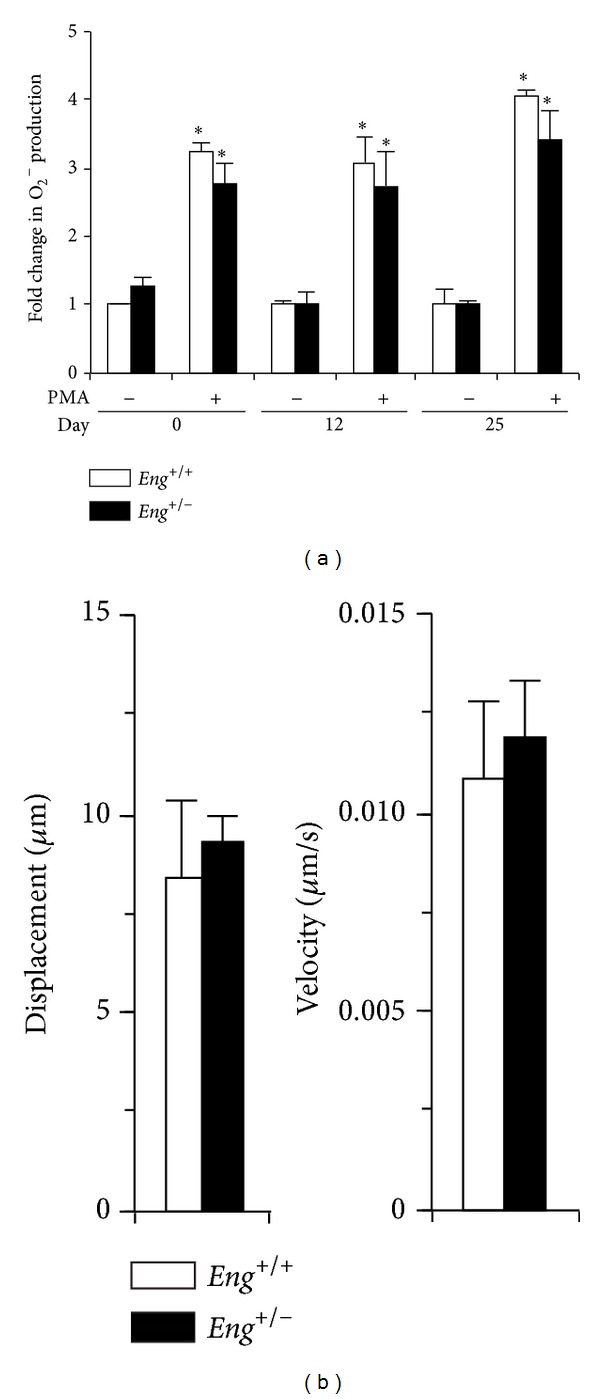
*Eng* ^+/−^ mice show normal bone marrow-derived neutrophil function. (a) Superoxide (O_2_^−^) production, measured by a dihydroethidium (DHE) based assay, was tested in isolated bone marrow-derived neutrophils from days 0, 12, and 25, with or without PMA stimulation. The results are plotted as fold change relative to the unstimulated* Eng* ^+/+^ control for each time point. *P < 0.05 versus corresponding unstimulated group. (b) Neutrophil migration capacity (displacement and velocity) was determined using fMLP as a chemotactic agent in both* Eng* ^+/+^ and* Eng* ^+/−^ mice. Results represent mean ± SEM (for DHE assay: *N* = 4–8 samples for days 0, 12, and 25; for chemotaxis assay: *N* = 3 experiments for each genotype).

As MPO and Nox-2 are expressed by neutrophils [39], the function of bone marrow neutrophils was tested in* Eng* ^+/+^ and* Eng* ^+/−^ mice. Neutrophils derived from normal and colitic mice demonstrated a similar capacity in both genotypes to produce superoxide (O2^−^) in response to phorbol 12-myristate 13-acetate (PMA) stimulation (Figure 5(a)). Furthermore, the migratory capacity of bone marrow derived neutrophils in response to* N*-formyl-methionyl-leucyl-phenylalanine (fMLP) stimulation was measured. Overall displacement and velocity of* Eng* ^+/−^ neutrophils were unimpaired (Figure 5(b)). Thus, these findings imply no intrinsic defect in bone marrow neutrophil ROS production and motility in* Eng* ^+/−^ mice.

## 4. Discussion

Our data show that the development of persistent inflammation in DSS-treated* Eng* ^+/−^ mice is marked by changes in relative bacterial content and by reduced expression of several factors of the TGF-*β* superfamily. Decreased levels of active TGF-*β*1, a key regulator of inflammation and of its activator, TSP-1 were observed in the colon of* Eng* ^+/−^ mice. In addition reduced expression of follistatin, known to counteract the proinflammatory effects of activins and regulate wound healing, was observed in colitic* Eng* ^+/−^ versus control mice. Enhanced expression of the neutrophil chemokine CXCL1 also correlated with a heightened inflammatory response occurring as early as the acute phase in colitic* Eng* ^+/−^ mice. Despite increased inflammation and myeloid infiltration, a gut-specific reduction in key myeloid ROS-generating enzymes was observed. These findings suggest that endoglin is required for resolution of inflammation and fully functional myeloid cells.

TGF-*β*1 is a potent inhibitor of immune activation and a promoter of wound healing [5, 8]. TGF-*β*1 is secreted in a latent form that requires posttranslational modifications to enable activation [7]. Here we report that endoglin haploinsufficiency is associated with lower levels of active TGF-*β*1 leading to impaired resolution in colitic* Eng* ^+/−^ mice. This reduction in active TGF-*β*1 corresponded with lower expression of TSP-1, a matricellular protein that activates latent TGF-*β*1 [26]. TSP-1 is known to have potent anti-inflammatory and angiostatic effects. Interestingly, mice deficient in TSP-1 showed expanded and prolonged heart postinfarction inflammation [41]. The mechanisms responsible for the anti-inflammatory effects of TSP-1 are still to be determined but may involve reduced levels of active TGF-*β*1 [42], as observed in our studies. TSP-1 could also be acting at the level of myeloid cells as it promotes clearance of apoptotic cells by macrophages [26]. In addition, TSP-1 is a potent angiostatic factor [43], and its reduction may contribute to the pathological (increased) angiogenesis observed in the colitic* Eng* ^+/−^ mice. Recent studies have also shown that endoglin regulates TSP-1 expression in endothelial cells [44, 45]. Thus, reduced TSP-1 levels and subsequent TGF-*β*1 activation in colonic tissue of* Eng* ^+/−^ mice could contribute to both sustained inflammation and pathological angiogenesis.

Colitic* Eng* ^+/−^ mice showed decreased levels of FST, which antagonizes activins and their induction of proinflammatory responses [46]. Activins are upregulated in inflammatory diseases, including IBD, and stimulate macrophage production of IL-1*β* and IL-6 [6, 46]. Furthermore, in several IBD models, administration of exogenous FST led to decreased disease severity with some improvement in epithelial repair [47]. Thus, the impaired wound healing in colitic* Eng* ^+/−^ mice could also be attributed to lower FST levels. In addition,* Eng* ^+/−^ mice demonstrated reduced expression of BMPER, which regulates BMP pathways, known to be modulated by endoglin. BMPER, which is expressed by endothelial cells, directly binds to various BMPs including BMP4 and BMP9 and inhibits their signaling [48, 49].* Bmper* ^+/−^ mice develop a proinflammatory endothelial phenotype due to increased expression of ICAM-1 and VCAM-1 [27, 28, 49]. Thus, the downregulation of BMPER in colitic* Eng* ^+/−^ mice may have enhanced endothelial cell activation, enabling increased leukocyte extravasation and sustained inflammatory response.

During the acute phase of colitis, the expression of several myeloid regulating cytokines/chemokines peaked in both genotypes, with higher levels of CXCL1 in colitic* Eng* ^+/−^ mice. As a result, there was increased infiltration of macrophages and a trend for more neutrophils in colonic LP of* Eng* ^+/−^ mice. In addition to being a proinflammatory factor, CXCL1 promotes angiogenesis [50, 51] and likely contributed to the dysregulated microvascular expansion in* Eng* ^+/−^ mice. During the resolution phase, both groups of mice demonstrated low levels of G-CSF, CXCL1, IL-6, and CCL11 relative to the acute phase of disease, but still higher than basal levels. However, in a related study with a larger sample size, we observed significantly higher G-CSF in* Eng* ^+/−^ mice undergoing persistent inflammation [52]. In that study, we also demonstrated higher levels of CCL2 (MCP-1) in colitic* Eng* ^+/−^ mice, which likely caused the increased monocyte/macrophage infiltration. Interestingly, antiangiogenic therapy led to reduced expression of G-CSF, supporting the concept of immune-driven angiogenesis contributing to persistent inflammation in colitic* Eng* ^+/−^ mice.

Despite increased myeloid infiltration, we observed heightened relative abundance of* C. coccoides* in the gut of* Eng* ^+/−^ mice showing persistent inflammation. Alterations in the abundance of this bacterial group have also been reported in IBD patients [53]. Furthermore, it has been suggested that immune dysfunction leads to changes in bacterial composition in IBD [1]. Reports of increased incidence of infection in HHT patients have also implicated several bacterial species [18, 19]. While these infections were attributed to poor blood filtration, alterations in immune responses could also be involved.

During inflammation, macrophages and neutrophils produce large amount of ROS, which oxidize proteins and lipids to eliminate infectious microbes [39]. The phagocyte NADPH oxidase complex is essential for efficient microbial killing by producing superoxide ions that are rapidly dismutated into H_2_O_2_. Impairment in phagocyte respiratory burst can lead to inefficient bacterial and fungal clearance, as observed in chronic granulomatous disease, where 65% of patients have Nox-2 mutations [54]. In the acute phase of colitis,* Eng* ^+/−^ and wild-type mice showed a similar increase in Nox-2; however, as colitis progressed, Nox-2 expression could not be sustained in* Eng* ^+/−^ mice, suggestive of exhaustion of the immune response. This was reflected by the lack of increase in H_2_O_2_ levels in colitic* Eng* ^+/−^ mice and may have contributed to the changes in microbiota composition. We could rule out an intrinsic bone marrow defect, as total Nox-2 levels and ROS production by neutrophils were not defective in* Eng* ^+/−^ mice.

MPO, which is primarily found in the azurophilic granules of neutrophils, is another enzyme that participates in the ROS-dependent microbicidal system by converting H_2_O_2_ and halides to hypohalous acids [39]. This enzyme is also present in macrophages [55]. Colonic MPO expression and activity were elevated in* Eng* ^+/+^ mice throughout colitis but decreased with time in DSS-treated* Eng* ^+/−^ mice. As Nox-2 provides superoxide and subsequently H_2_O_2_, reduction of both Nox-2 and MPO would have an additive effect on decreasing bactericidal activity in colitic* Eng* ^+/−^ mice. These defects were not observed in the bone marrow of* Eng* ^+/−^ mice but were rather gut specific and perhaps influenced by the inflammatory environment in the colon. Studying the direct effects of endoglin in these gut neutrophils and macrophages was not possible as leukocyte isolation from the colon led to significant cell loss. More recently, a myeloid-specific endoglin knockout model has been generated [56], which exhibits increased incidence of sporadic infections. This model will allow the determination of direct effects of myeloid endoglin on inflammation and its resolution.

Overall, we have shown that persistent inflammation in* Eng* ^+/−^ mice is associated with altered expression of factors that regulate inflammation, including TGF-*β*1, TSP-1, FST, BMPER, and CXCL1. We show that myeloid recruitment to the inflamed gut is increased in DSS-treated* Eng* ^+/−^ mice and that endoglin may regulate myeloid-mediated ROS production. These potential novel roles for endoglin may be important for understanding the resolution of inflammation and the higher incidence of infections in HHT patients.

## Figures and Tables

**Table 1:** Expression of cytokines and chemokines in the distal colon during DSS-induced colitis in *Eng* ^+/+^ and *Eng* ^+/−^ mice.

Cytokine	Cytokine levels (pg/mg tissue protein)					
	*Eng* ^ +/+^			*Eng* ^ +/−^		
	Day 0	Days 7–9	Days 18–23	Day 0	Days 7–9	Days 18–23
CXCL1	10.9 ± 0.6	791 ± 196*	49 ± 8.8*	11.6 ± 2.3	1614 ± 261^∗†^	138 ± 75*
G-CSF	1.3 ± 0.3	3319 ± 1028*	35 ± 8.0*	1.2 ± 0.3	5740 ± 1209*	100 ± 49*
IL-6	2.3 ± 0.2	1516 ± 569*	35 ± 10*	2.6 ± 0.2	2608 ± 735*	220 ± 142*
CCL11	45 ± 3.5	514 ± 77*	99 ± 6.8*	58 ± 9.2	638 ± 45*	119 ± 20^#^
Il-1*β*	13.0 ± 2.3	66 ± 16*	20 ± 1.7	13.5 ± 0.7	51 ± 13*	42 ± 20
IL-10	9.2 ± 2.0	37 ± 13*	24 ± 5.1	8.0 ± 2.3	24 ± 2.7*	18 ± 3.7
TNF*α*	1.9 ± 0.7	12.0 ± 2.7*	2.4 ± 0.9	1.8 ± 0.9	5.8 ± 0.8^∗†^	3.0 ± 0.9
IFN*γ*	3.4 ± 0.6	20 ± 10.6	6.0 ± 1.4	2.7 ± 0.1	4.8 ± 1.6	9.2 ± 4.3
IL-17	1.1 ± 0.3	7.0 ± 2.6	2.6 ± 0.6	1.0 ± 0.2	2.0 ± 0.6	5.3 ± 2.1
M-CSF	1.5 ± 0.6	9.4 ± 4.3	1.2 ± 0.4	1.5 ± 0.7	25 ± 15	0.8 ± 0.2
GM-CSF	8.0 ± 1.5	8.4 ± 2.2	6.6 ± 2.5	8.6 ± 3.5	8.3 ± 2.5	3.7 ± 1.8
IL-12p40	6.1 ± 1.5	4.0 ± 1.2	3.8 ± 0.9	6.2 ± 1.4	1.8 ± 0.7	2.0 ± 0.6
IL-12p70	0.8 ± 0.4	1.1 ± 0.4	0.3 ± 0.2	1.2 ± 1.0	1.0 ± 0.4	0.4 ± 0.4
IL-4	1.4 ± 0.1	1.7 ± 0.1	1.4 ± 0.1	1.6 ± 0.1	1.4 ± 0.1	1.3 ± 0.1

Results represent mean ± SEM (for both genotypes, *N* = 4 mice for day 0, *N* = 7-8 mice for days 7–9 and 18–23). *P < 0.05 versus corresponding day 0, ^#^ *P* = 0.06 versus corresponding day 0, and ^†^ *P* < 0.05 versus corresponding *Eng* ^+/+^.

**Table 2:** Distribution and number of major colonic leukocytes during DSS-induced colitis in *Eng* ^+/+^ and *Eng* ^+/−^ mice.

Experimental day(s)	Percent CD45^+^ cells					
	B cells		T cells		Myeloid cells	
	*Eng* ^ +/+^	*Eng* ^ +/−^	*Eng* ^ +/+^	*Eng* ^ +/−^	*Eng* ^ +/+^	*Eng* ^ +/−^
0	31 ± 5	35 ± 5	14 ± 1	13 ± 1	22 ± 3	19 ± 2
7–9	33 ± 1	35 ± 8	14 ± 1	14 ± 1	37 ± 1*	32 ± 8
18–23	33 ± 2	26 ± 4	16 ± 1	16 ± 1	23 ± 1	30 ± 5^#^

	Total number of cells (×10^6^)					
	B cells		T cells		Myeloid cells	

0	0.4 ± 0.1	0.5 ± 0.1	0.2 ± 0.01	0.2 ± 0.01	0.3 ± 0.02	0.3 ± 0.04
7–9	0.9 ± 0.1	1.6 ± 0.4	0.5 ± 0.1	0.6 ± 0.1	1.2 ± 0.2	1.5 ± 0.3
18–23	2.5 ± 0.3*	2.3 ± 0.4*	1.2 ± 0.2*	1.5 ± 0.2*	1.8 ± 0.2*	2.9 ± 0.8*

Results represent mean ± SEM (for both genotypes, *N* = 3 experiments for day 0, 5 for days 7–9 and 4 for days 18–23, with 3-4 mice/experiment). *P < 0.05 versus corresponding day 0, ^#^ *P* = 0.08 versus corresponding day 0.
